# Consensus and Controversy in the Debate over the Biphasic Impact of Alcohol Consumption on the Cardiovascular System

**DOI:** 10.3390/nu13041076

**Published:** 2021-03-25

**Authors:** Cristian Stătescu, Alexandra Clement, Ionela-Lăcrămioara Șerban, Radu Sascău

**Affiliations:** 1 Internal Medicine Department, “Grigore T. Popa” University of Medicine and Pharmacy, 700503 Iași, Romania; cstatescu@gmail.com (C.S.); radu.sascau@gmail.com (R.S.); 2 Cardiology Department, Cardiovascular Diseases Institute “Prof. Dr. George I.M. Georgescu”, 700503 Iași, Romania; 3 Physiology Department, “Grigore T. Popa” University of Medicine and Pharmacy, 700503 Iași, Romania; ionela.serban@umfiasi.ro

**Keywords:** alcohol, cardiovascular disease, risk factor, amount of consumption

## Abstract

In the past few decades, research has focused on the importance of addressing modifiable risk factors as a means of lowering the risk of cardiovascular disease (CVD), which represents the worldwide leading cause of death. For quite a long time, it has been considered that ethanol intake has a biphasic impact on the cardiovascular system, mainly depending on the drinking pattern, amount of consumption, and type of alcoholic beverage. Multiple case-control studies and meta-analyses reported the existence of a “U-type” or “J-shaped” relationship between alcohol and CVD, as well as mortality, indicating that low to moderate alcohol consumption decreases the number of adverse cardiovascular events and deaths compared to abstinence, while excessive alcohol use has unquestionably deleterious effects on the circulatory system. However, beginning in the early 2000s, the cardioprotective effects of low doses of alcohol were abnegated by the results of large epidemiological studies. Therefore, this narrative review aims to reiterate the association of alcohol use with cardiac arrhythmias, dilated cardiomyopathy, arterial hypertension, atherosclerotic vascular disease, and type 2 diabetes mellitus, highlighting literature disagreements over the risk and benefits of low to moderate drinking on the cardiovascular system.

## 1. Introduction

In the past few decades, research has focused on the importance of addressing modifiable risk factors as a means of lowering the risk of cardiovascular disease (CVD), which represents the worldwide leading cause of death [1]. Alcohol intake typifies a well-known trigger of CVD and the dominant cause of death in males aged 15 to 59 years [2]. 

For quite a long time, it has been considered that ethanol intake has a biphasic impact on the cardiovascular system, mainly depending on the drinking pattern, amount of consumption, and type of alcoholic beverage [3]. Multiple case-control studies and meta-analyses reported the existence of a “U-type” or “J-shaped” relationship between alcohol and CVD, as well as mortality, indicating that low to moderate alcohol consumption decreases the number of adverse cardiovascular events and deaths compared to abstinence, while excessive alcohol use has unquestionably deleterious effects on the circulatory system [4].

However, beginning in the early 2000s, the cardioprotective effects of low doses of alcohol were abnegated by the results of large epidemiological studies [5]. It is difficult to appreciate if there is a causal relationship between alcohol intake and CVD endpoints, as most of the evidence originates in observational studies, and only randomized clinical trials would provide decisive information over the protective role of low to moderate alcohol consumption on cardiovascular outcomes. Therefore, the Moderate Alcohol and Cardiovascular Health Trial (MACH I 5) is a multicenter randomized trial to firstly evaluate the beneficial effect of 15g of alcohol daily compared to abstention on 7800 subjects with enhanced cardiovascular risk, aged 50 years or above, followed up for six years. It aims to recognize, with >80% power, a 15% decrease in the risk of non-fatal myocardial infarction, non-fatal ischemic stroke, hospitalized angina, coronary/carotid revascularization, or total mortality and secondarily of diabetes [6].

The 2015 U.S. Dietary Guidelines for Americans strongly suggest restricting consumption to ≤2 drinks/day for men and ≤1 drink/day for women [7]. Of note, alcohol intake is reported as either grams per day or standard drinks. A standard drink contains 12–15 g of pure ethanol and corresponds to 100–125 mL 12% alcohol by volume wine, 240–300 mL 5% alcohol by volume beer, or 30–37.5 mL 40% alcohol by volume spirits. Heavy drinking is defined as a long-term, high-dose intake, of >60 g/day in men and >40 g/day in women [8]. Conversely, binge drinking describes a short-term consumption pattern, of >4–5 drinks per occasion, affecting more than 38 million adults from the United States [9]. Except for self-reported alcohol use, urinary ethyl glucuronide can be used as a reliable tool in assessing habitual ethanol drinking, with good correspondence to the risk of CVD [10].

**Figure 1:**
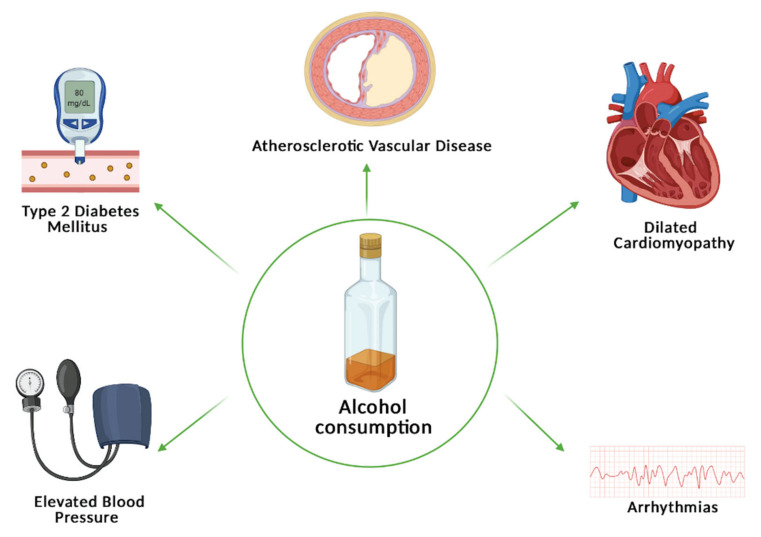
The association between alcohol consumption and arrhythmias, dilated cardiomyopathy, hypertension, atherosclerotic vascular disease, and type 2 diabetes mellitus.

In the light of the abovementioned data, this narrative review aims to reiterate the association of alcohol use with cardiac arrhythmias, dilated cardiomyopathy, arterial hypertension, atherosclerotic vascular disease, and type 2 diabetes mellitus, highlighting literature disagreements over the risk and benefits of low to moderate drinking on the cardiovascular system (Figure 1).

A literature search on several databases was conducted, including MEDLINE, PUBMED, and EMBASE. Clinical Evidence, UpToDate, and the US ClinicalTrials.gov Registry (accessed on 15 February 2021) were also revised. The following search terms were used: alcohol consumption; ethanol intake; cardiovascular disease; alcoholic cardiomyopathy; holiday heart syndrome; and alcohol-related arrhythmias. We selected publications from 1978 to 2021 (case reports, reviews, meta-analyses, case studies, comparative studies, human observational studies, animal and in vitro studies) that evaluated the relationship between alcohol use and cardiovascular diseases. The references of the initially selected articles, as well as the complete text papers, were also analyzed and introduced, when relevant.

## 2. Background

The Prospective Urban Rural Epidemiology (PURE) study evaluated the association between 14 potentially modifiable risk factors, including alcohol use and CVD and mortality in 155,722 subjects from 21 different countries grouped by economic levels. It has been estimated that approximately 70% of CVD and death cases are due to modifiable risk factors. Within the analysis, 29% of the overall study population reported drinking alcohol, high-income countries displaying higher consumption rates [11].

By implementing a 2-sample Mendelian randomization design and analyzing data of genome-wide association studies, including more than 1.2 million participants, Rossof et al. failed to prove a genetic basis for a cardioprotective effect of ethanol consumption on CVD risk factors and outcomes. This research supports the increasing evidence that the beneficial impact of low to moderate-alcohol intake previously reported by several studies might be the results of concomitant healthier lifestyles [12]. 

Addedly, a large-scale population-based cohort study of 1.93 million participants with no evidence of CVD at enrolment proved that high alcohol intake is connected to a decreased risk of an initial acute myocardial infarction. Still, it increases the risk of other alcohol-related CVD, including unheralded coronary death, heart failure, cardiac arrest, transient ischemic attack, ischemic stroke, intracerebral hemorrhage, and peripheral arterial disease, as well as all-cause mortality. Most importantly, in this comprehensive analysis, the authors emphasized that moderate drinking is linked to a lower risk of first addressing with several, but not all CVDs, further endorsing the controversial effects of alcohol on the cardiovascular system [13]. 

A study from the National Health Interview Surveys, comprising a total of 152,180 subjects from the United States of America, assessed the relationship between ethanol drinking and the risk of all-cause mortality. In this analysis, light and moderate drinking were linked with a lower risk of all-cause mortality in whites but not in Blacks, thus emphasizing the high variability of alcohol effects among different races [14]. Ethnicity and culture appear to be of additional contribution, previous reports failing to prove a cardioprotective effect of light and moderate consumption on health outcomes in Chinese and Indians [2,15,16].

Later, based on a 30-year cohort study, including a total of 59,312 study participants, Jankhotkaev and coworkers failed to prove a J-shaped curve between ethanol intake and all-cause mortality and reported that regular alcohol consumption is linked with increased all-cause and cancer mortality [17].

## 3. Alcohol-Related Arrhythmia

The “Holiday Heart Syndrome” was described in the early 1970s by Philip Ettinger. He reported for the first time the link between binge drinking and acute heart rhythm disturbances, especially atrial fibrillation [18]. The term was employed to describe: “acute arrhythmias precipitated by excessive alcohol intake in subjects with no clinical evidence of structural heart disease, that disappeared with alcohol withdrawal” [19,20]. The holiday heart syndrome nominates a common reason for hospital emergency department visits, up to 35 to 62% of atrial fibrillation cases being induced by alcohol abuse [21,22,23]. As underlined by the MunichBREW study results that included 3028 volunteers who attended the 2015 Munich Octoberfest festival, sinus tachycardia is another common arrhythmia related to acute alcohol consumption authors naming autonomic imbalance as a major trigger of supraventricular arrhythmias [24].

At a molecular level, binge alcohol exposure seems to exert its proarrhythmic effect via the activation of atrial c-Jun-N-terminal kinase (JNK). Activated JNK 2 promotes the phosphorylation of calmodulin kinase II (CaMKII), with further changes in the intracellular Ca^2+^ dynamics, abnormal Ca^2+^ waves, and increased predisposition to atrial arrhythmia [9].

A recent study including 107,485 subjects followed up for nearly 14 years found that the consumption of only 12 g ethanol per day was linked to a 16% increase in the risk of developing atrial fibrillation, regardless of the beverage type. This finding adds to the growing body of evidence that even small amounts of alcohol intake favor CVD development, including atrial fibrillation [25].

**Figure 2:**
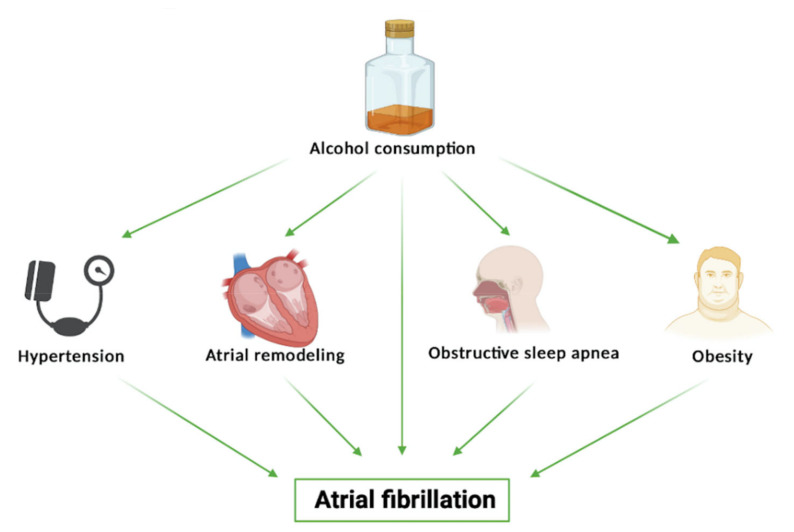
The pathophysiology of alcohol-related atrial fibrillation.

Nevertheless, heavy alcohol drinking is recognized to be linked with new-onset atrial fibrillation. The pathophysiological mechanisms linking chronic alcohol abuse and atrial fibrillation are multiple. Long-term consumption generates progressive atrial remodeling and favors the development of obesity, hypertension, and sleep-disordered breathing, well-known promoters of new-onset atrial fibrillation (Figure 2) [21]. 

According to a recent nationwide population-based study that included 9,776,956 patients from the Republic of Korea, the frequency of drinking and the amount of alcohol consumption per week significantly correlate with the risk of developing atrial fibrillation. Surprisingly, in the analysis performed by Kim et al., the ingestion of large amounts of alcohol per drinking session, known as binge drinking, did not influence the rate of new-onset of atrial fibrillation [26].

In what concerns the clinical significance of alcohol intake in various age groups, another nationwide Korean populational study, comprising 9,797,409 subjects without a prior diagnosis of atrial fibrillation, highlighted that heavy drinkers have a substantial risk of developing atrial fibrillation if aged 30 years or above. No meaningful consideration was noticed in the 20s, while mild- to moderate drinking increased the susceptibility to atrial fibrillation in subjects aged 60 years or above [27].

Although the effect of regular alcohol consumption on incident atrial fibrillation has been largely studied, little is known about the impact of cessation on secondary prevention of atrial fibrillation. In this respect, Voskoboinik et al. performed a multicenter, prospective, open-label, randomized, controlled-trial, comprising a total number of 140 patients that consumed 10 or more standard drinks per week and had a history of paroxysmal or persistent atrial fibrillation. All subjects were in sinus rhythm at the time of the inclusion and were randomly assigned in a 1:1 ratio to either the abstinence or the control group. During 6 months of follow-up, the recurrence rate, as well as the burden of atrial fibrillation, were lower in the abstinence group, thus emphasizing the value of lifestyle modification and remarkably of alcohol withdrawal [28].

Despite being deemed a benign condition, emerging evidence indicates that holiday heart syndrome can precipitate sudden cardiac death, especially in subjects with a CVD history [21]. Out of 5869 sudden cardiac victims included in the Fingesture cohort, in 290 cases (4.9%), the underlying cause of death was alcoholic cardiomyopathy [29]. 

Alcohol intake determines QT interval prolongation, which in turn predisposes to potentially malignant ventricular arrhythmias, irrespective of the presence of coronary artery disease (CAD) [30]. In a case-crossover study design, including 309 subjects who died of sudden cardiac death, with 2.8 risk factors for CAD, the relative risk of dying within 2 h after ingesting alcohol was 3.00 [31]. 

All the aforementioned information demonstrates ethanol’s potency to initiate and maintain cardiac arrhythmias, independently of the quantity and type of consumption.

## 4. Alcoholic Cardiomyopathy

**Figure 3:**
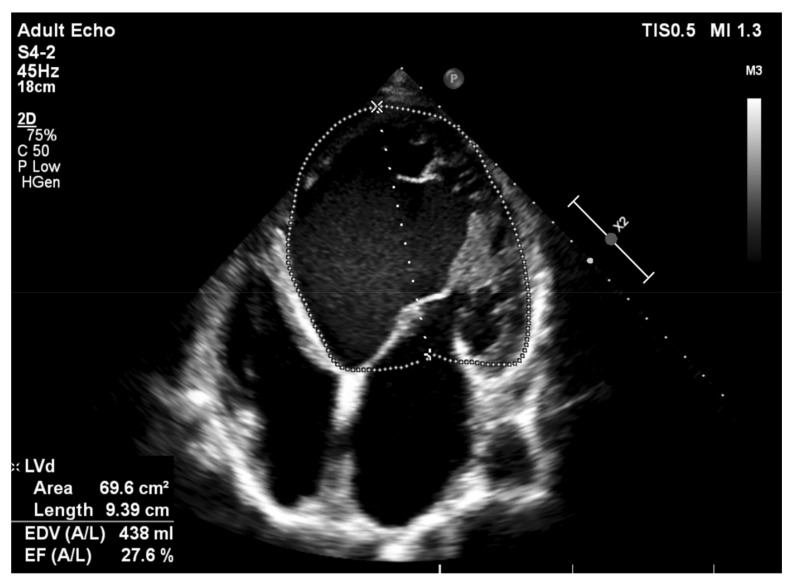
Transthoracic echocardiography—apical 4-chamber view: severe left ventricular dilation with markedly decreased ejection fraction in a 47-year-old subject with a history of long-term alcohol abuse (authors’ personal archive).

Long-term alcohol excessive consumption results in alcoholic cardiomyopathy (ACM), which is a secondary nonischemic dilated cardiomyopathy and the most frequent type of ethanol-induced heart damage [32,33]. ACM is characterized by left ventricular dilation with impaired systolic function, and its emergence has been closely linked with the quantity and total time of ethanol intake. Figure 3 shows the transthoracic echocardiographic evaluation of a 47-year-old subject with a 20-year history of alcoholism (approximately 16 standard drinks per day). In this patient, the apical 4-chamber view illustrates severe left ventricular enlargement, with global systolic impairment (an ejection fraction assessed via the biplane Simpson’s method of 27%). Research has shown that ACM occurs even in the absence of protein or caloric malnutrition, thus demonstrating that ethanol causes ACM in a cumulative dose-dependent manner, irrespective of other metabolic deficiencies, but highly dependent on the total lifetime dose of ethanol [34]. A consumption of >80 g/day for a minimum of 5 years represents an independent risk factor for ACM development, greater lifetime exposure conducive to higher risks, and women displaying lower thresholds [35,36]. 

A significant body of research suggests that there is an underestimated genetic contribution in ACM and that genetic testing should be performed in all ACM subjects. Titin truncating variants (TTNtv) promote the appearance of ACM, and the coexistence of TTNtv and excessive alcohol consumption leads to a worse left ventricular ejection fraction in dilated cardiomyopathy, as stated by Ware et al. [37].

The phenotypes of dilated and alcoholic cardiomyopathies are overlapping, and there is a paucity of data regarding the clinical, immunological, and biological features of these two entities. On this basis, Li et al. performed a retrospective analysis that aimed to assess the histopathologic characteristic of ACM when compared to idiopathic dilated cardiomyopathy and noticed no significant difference in the amount of left ventricular interstitial fibrosis. However, they suggested that there might be a difference in overall myocyte ratio between these two pathologies [38].

Additionally, limited information is available concerning the natural history, outcomes, and long-term prognosis of ACM. Among 75,430 ACM hospital stays registered in the National Inpatient Sample (NIS) database from 2007 to 2014, cardiac arrhythmias complicated 48.2% of hospitalizations and represented the primary discharge diagnosis in 10.6%. The high burden of arrhythmias was mainly attributable to atrial fibrillation/atrial flutter and did not result in increased in-hospital mortality, but rather in more extended hospital stays and higher total charges [39]. 

Similarly, Ram et al. sought to evaluate the trends in hospitalizations and ACM outcomes by analyzing the Nationwide Inpatient Sample’s discharge data from 2002 through 2014. Their final study population consisted of 45,365 ACM subjects, and within the research, there were four significant findings, as follows: a slight decrease in the number of ACM-related hospitalizations, a large variation through the years in the percentage of in-hospital mortality without the identification of any up/down pattern, an increase in the proportion of patients pertaining to the Caucasian race as well as in the prevalence of other comorbidities including smoking, drug abuse, depression, and hypertension, and the recognition of acute heart failure as the most frequent cause of hospital admission [40].

According to a recent, retrospective, observational study of 321 ACM subjects, followed up for a mean period of 3.78 years, QRS complex duration on electrocardiogram, systolic blood pressure, and New York Heart Association class at admission significantly connects with all-cause mortality [41]. 

As there are no safe amounts of alcohol intake in ACM, abstinence is highly recommended and helps to optimize the medical treatment, mainly consisting of beta-blockers, angiotensin converting enzymeinhibitors, and diuretics. Alcohol withdrawal can lead to complete cardiac recovery when ACM is diagnosed in an early stage [19]. 

## 5. Alcohol Intake and Elevated Blood Pressure

Hypertension is a major public health issue, accounting for substantial mortality and morbidity worldwide, being defined by a resting systolic blood pressure (BP) higher than 140 mmHg and/or a resting diastolic BP higher than 90 mmHg. It either possesses a genetic background or is related to behavioral risk factors, such as obesity, sedentary lifestyle, smoking, and alcohol abuse [42]. The differences in the burden of risk factors for elevated blood pressure partially explain the heterogeneity in the prevalence and distribution of hypertension, the leading cause of CVD and premature death worldwide [43]. According to the research of Zhao et al., who analyzed longitudinal data from the China Health and Nutrition Survey, comprising 50,013 records of 12,577 subjects, high drinking frequency leads to a high prevalence of hypertension, both in men and women [44].

Moreover, as emphasized by numerous, large-scale, population-based epidemiological studies, lifestyle modification, including reducing the average alcohol consumption, is the mainstay for hypertension prevention and control. 

In a cross-sectional study comprising more than 2.7 million adult primary care patients, unhealthy alcohol consumption was communicated in 269,379 subjects, with higher odds of exceeding daily and weekly recommended amounts recorded among hypertensive subjects [45]. 

The Cochrane Hypertension Information Specialist sought to assess the short-term dose-related effects of alcohol on systolic and diastolic BP in healthy and hypertensive subjects aged over 18 years when compared to placebo. According to their results, high alcohol intake has an acute biphasic impact on BP, decreasing BP within the first 6 to 12 h after intake, followed by an increase at more than 13 h after consumption [46].

As with other cardiovascular diseases, chronic ethanol intake was initially considered to increase BP in a gender-specific, “J-shaped” pattern, dose-dependent manner [2]. However, a recent meta-analysis of Roerecke et al. failed to prove any protective effect of small amounts of alcohol consumption in women and highlighted that any drinking level increases the risk of hypertension among men [47]. Similar to these findings, a systematic review and meta-analysis performed by Jung and coworkers sought to evaluate the effect of alcohol dose on hypertension incidence among Asian and Western men. Within the analysis, 11 articles were selected, 7 Asian and 4 Western, and a dose–response relationship was detected between ethanol consumption and blood pressure values. These results epitomize further evidence that even low alcohol quantities promote the development of arterial hypertension [48].

The Action to Control Cardiovascular Risk in Diabetes (ACCORD) trial sought to assess whether cardiovascular risk could be decreased in diabetic subjects via three major therapeutic strategies, as follows: rigorous glycemic control, strict blood pressure control, and tight lipid control. When analyzing the relationship between alcohol consumption and blood pressure control in 102,000 eligible participants of the ACCORD study, it was revealed that not only heavy but also moderate ethanol intake is linked with hypertension and poor cardiovascular outcomes in patients with diabetes mellitus [49]. 

Therefore, restraint of alcohol intake is a class I level of evidence A recommendation in the current European guidelines for the management of arterial hypertension [50]. With adequate therapeutic intervention, screening of alcohol abuse might help to lower BP levels and has already been implemented in different European countries [51]. However, time constraints, stigma, and underscored significance of alcohol consumption seem to be major screening hindrances [52].

Additionally, based on the results of a survey study conducted in 2015, on 1064 physicians attending annual medical meetings, the lack of self-reported alcohol screening among subjects with newly diagnosed and treatment-resistant hypertension represents a critical shortage on hypertension management. Even though there is an increased awareness about alcohol-associated elevated blood pressure, the percentage of general practitioners screening for alcohol consumption in these two categories of patients is relatively low [53]. 

## 6. Alcohol Consumption and Atherosclerotic Vascular Disease

Coronary artery disease (CAD) or coronary heart disease (CHD) has been repeatedly categorized as the primary cause of death and disability worldwide, its prevalence and severity being closely related to that of lifestyle risk factors [54]

Due to the multifactorial nature of atherosclerosis, it is difficult to estimate the alcohol-attributable atherosclerosis burden. However, out of 7350 subjects with a recent CHD event, included in the European Society of Cardiology—EURObservational Research Program (ESC-EORP) European Action on Secondary and Primary Prevention by Intervention to Reduce Events (EURO-ASPIRE) survey, 47% men and 23% women reported alcohol consumption [55].

The importance of addressing sociobehavioral risk factors as a means of improving outcomes in CAD was supplementarily strengthened by the findings of Bortnick et al., who analyzed 1208 individuals from a low-income urban setting, presenting with an ST- segment elevation myocardial infarction between 2008 and 2014. A high burden of risk factors was documented within the study population, alcohol consumption being more frequently recorded among middle-aged men, and age-matched women displaying higher rates of metabolic disorders [56]. 

Analogously to the previous study design, Gaudel P et al. aimed to assess the risk profile of 224 CHD subjects from Nepal in a cross-sectional analysis. By using standard questionnaires, information regarding eating habits, smoking, ethanol intake, perceived stress, physical activity, body weight, and compliance to therapeutic regimens was attained. Even though there was a high prevalence of self-reported risk factors within the study cohort, on this occasion, alcohol consumption was reported by only 23% of participants [57].

Nevertheless, heavy drinking increases the risk of CHD mortality both in women and men, apart from the enhanced risk of stroke, either hemorrhagic or ischemic related to alcohol consumption [58]. 

The European Prospective Investigation into Cancer and nutrition cohort (EPIC-CVD) study aimed to evaluate the connection between ethanol intake and the risk of incident non-fatal and fatal CHD and stroke. Only individuals without CVD at baseline, from eight European countries, were recruited in the analysis. Both baseline and lifetime alcohol consumption were inversely connected to non-fatal CHD risk and positively linked to the risk of stroke. In what concerns the risk of fatal CHD, a J-shaped relationship with baseline ethanol intake was described this time [59]. 

The atherosclerosis profile assessed via coronary computed tomography angiography appears slightly different in subjects with end-stage liver disease due to excessive alcohol consumption. As stated by Steinkohl F., high-risk atherosclerotic plaques with lipid-rich core are less frequent among patients with alcohol-related end-stage liver disease when compared to healthy controls, this population exhibiting a greater fibrocalcified plaque burden, though with high overall CHD prevalence. Recurrent episodes of elevated blood pressure occasioned by heavy drinking appear to favor coronary calcification [60]. Regarding the coronary artery diameter, no significant difference exists between heavy, moderate, and non-drinkers, independently of the smoking status, as indicated by Yang and coworkers’ prospective analysis [61].

In what concerns the linkage between alcohol consumption and peripheral vascular disease, according to the results of a single-center prospective study including 342 subjects with more than one cardiovascular risk factor or confirmed CVD, ethanol intake positively correlates with peripheral atherosclerotic plaque volume as assessed via three-dimensional ultrasound imaging [62]. Furthermore, a dose–response relationship between alcohol consumption and diabetic lower extremity arterial disease was documented among 138 Chinese alcohol users with type 2 diabetes mellitus when compared to 833 diabetic subjects without a drinking history [63]. The early impact of behavioral risk factors on arterial health was documented by the ALSPAC study, which evaluated the effects of smoking and alcohol use on arterial stiffness in 1266 young subjects. At 17 years, the carotid to femoral pulse wave velocity (PWV) was assessed, and it appeared that high-intensity drinking, defined as >10 drinks on a typical day, resulted in greater PWV values, smoking exposure having a complementary effect. Of note, no connection between the age of starting and the frequency of drinking and PWV was described. Nevertheless, this large British cohort study emphasized once more the need for timely public health interventions [64].

## 7. Linkage of Alcohol Consumption with Type 2 Diabetes Mellitus—A Notorious Cardiovascular Risk Factor

Frequent alcohol drinking increases the risk of metabolic syndrome and subsequently of diabetes [65]. Adherence to healthy lifestyle habits and thus a reduction in the maximum intake of alcoholic beverages prevents the development of type 2 diabetes mellitus (T2DM), a prime risk factor for CVD, and improves survival among diabetic subjects [66].

According to the results of a recent prospective multicohort study, a high overall healthy lifestyle score accounts for a significant increase in the years lived without major chronic disease. Within this analysis, four baseline lifestyle factors were considered: smoking, body mass index, physical activity, and alcohol consumption. Among the 116,043 recruited subjects, a body mass index lower than 25 kg/m^2^ and the coexistence of at least 2 of the following factors: never smoking, physical activity, and moderate alcohol consumption led to the highest number of years free of chronic pathologies, including T2DM and CHD [67]. The diabetes–alcohol consumption interaction was complementarily endorsed by a cross-sectional study of 803,164 individuals from India, where ethanol drinking was linked with increased odds of diabetes [68].

However, as with other CVD and risk factors, the dose–response relationship between alcohol consumption and T2DM is inconsistent. Numerous cohort studies and meta-analyses reported that light to moderate drinking is linked with a lower risk of T2DM. By contrast, a 10-year longitudinal data study including 2366 Koreans, aged 40–69 years, with no baseline evidence of T2DM and body mass index values < 23 kg/m^2^ suggested that the consumption of more than 2 units of alcohol per day increases the risk of T2DM and that abstinence might help to prevent the development of lean T2DM [69].

Additionally, the correlation between alcohol drinking and the risk of T2DM appears to be different in the presence of fatty liver disease (FLD). When assessing the combined effect of alcohol consumption and FLD on incident T2DM among 9948 men, Okamura et al. found that heavy alcohol drinkers with concomitant FLD have a higher risk of T2M. Interestingly, in this research, none or minimal alcohol consumption was linked with a reduced risk of incident T2DM in subjects without FLD, but not in those with FLD [70]. On the other hand, moderate alcohol consumption correlates with extensive fibrosis in non-alcoholic FLD, exhibiting a synergistic effect with T2DM [71]. 

At a molecular level, long-term alcohol intake induces fibroblast growth factor 21-mediated pancreatic islet dysfunction and apoptosis, enhancing the risk of diabetes [72].

It is essential to acknowledge that a delayed age of alcohol onset and a reduced drinking duration result in a decreased risk of T2DM, as emphasized by the largest prospective cohort of Chinese adults study, which comprised a total of 512,712 participants [73]. 

Table 1 summarizes the effects of high-dose ethanol versus low to moderate alcohol consumption on the risk of arrhythmias, dilated cardiomyopathy, hypertension, atherosclerotic vascular disease, and type 2 diabetes mellitus, as previously depicted.

## 8. Established Benefits of Dietary Interventions with Alcoholic Beverages

The Mediterranean diet was firstly characterized by Keys et al. in 1986, when it was emphasized that the subjects who adhere to this dietary pattern have a lower risk of cardiovascular disease than those who consume a typical Western diet [74]. Today, it is one of the most evaluated dietary patterns from the scientific literature, strong evidence indicating a lower risk of all-cause mortality, CVD, T2DM, cognitive disorders, and site-specific cancer with high adhesion to this recommendation [75,76]. Conventionally, this diet supports the consumption of large amounts of vegetables, fruits, nuts, grains, fish, seafood, poultry, and olive oil, along with low to moderate intake of wine, mainly red and especially during meals [75]. 

The light to moderate consumption of red wine has been suggested as the underlying explanation for the so-called “French Paradox”, the French population displaying a lower incidence of coronary heart disease and mortality despite consuming high amounts of saturated fats [77]. Various bioactive components of red wine have proven beneficial cardiovascular effects. A growing body of evidence indicates that red wine polyphenols (e.g., flavonols, flavanols, anthocyanin, and stilbenes) possess antioxidant, anti-inflammatory, antiatherosclerotic, and antithrombotic properties and improve lipid profile, insulin sensitivity, and endothelial function [78]. Among these polyphenolic compounds, resveratrol is considered the most effective in preventing coronary heart disease, exerting antidiabetic and antihypertensive effects [79]. It has also been postulated that resveratrol could present beneficial antiarrhythmic properties in atrial fibrillation via the inhibition of intracellular calcium release and pathological signaling pathways [80].

Likewise, in the Cardiovascular Diabetes and Ethanol (CASCADE) trial, 224 well-controlled diabetic subjects were randomly assigned to 150 mL of mineral water, white wine, or red wine with dinner for 2 years. The initiation of moderate wine consumption, especially red wine, in diabetic patients, was apparently safe and resulted in a moderate decrease of cardiometabolic risk [81]. The cardioprotective actions of moderate alcohol consumption are also related to the type of consumed alcoholic beverages, the reported benefits of other ethanolic drinks being inferior to those of red wine. Nevertheless, it appears that beer also exerts beneficial effects on cardiovascular health when consumed in low to moderate amounts. The non-alcoholic compounds of beer (kaempferol, quercetin, tyrosol, and phenolic acids) have also been linked to antioxidant, anti-inflammatory, and vasodilating effects, improving endothelial function and pressure wave reflections [82]. Additionally, in a randomized, crossover study that aimed to evaluate the cardiovascular implications of red wine versus vodka, increased levels of apolipoprotein A1 (an antiatherogenic molecule) were found only following vodka consumption. In this analysis, 85 healthy men were randomly assigned to consume either red wine or vodka (3 units/day) for two weeks. After a two-week wash-out period, subjects were given the alternate alcoholic drink for other 2 weeks. Out of 85 recruited participants, only 77 participants completed the study [83].

Therefore, further studies addressing the tangled relationship between low to moderate alcohol consumption and cardiovascular health are needed.

## 9. Conclusions

A comprehensive assessment of lifestyle risk factors and the implementation of proper interventional strategies represent fundamental pillars of cardiovascular disease prevention. There is an intriguing relationship between alcohol consumption and the risk of cardiovascular diseases. Although the harmful health consequences of heavy drinking have been consistently reported, low to moderate alcohol intake’s cardiovascular implications are still ambiguous. As previously described, this heterogeneous response to low to moderate alcohol exposure can be partly explained by race (light to moderate drinking being connected with a lower risk of all-cause mortality in whites, but not in Blacks), ethnic origin (Italians displaying improved health outcomes with low to moderate drinking, while the Chinese and the Indians do not), genetic background (the coexistence of titin truncating variants leading to a more severe alcoholic cardiomyopathy phenotype), and type of alcoholic beverage (red wine possessing antioxidant and anti-inflammatory properties, improving endothelial function and insulin resistance). Until further knowledge is attained via large randomized controlled trials, it is essential to recognize that regular alcohol use is a preventable risk factor for dilated cardiomyopathy, higher lifetime consumption leading to a greater risk of development. Both binge drinking and high lifetime ethanol consumption increase the risk of atrial fibrillation and other alcohol-related cardiac arrhythmias. Alcohol intake has detrimental effects on hypertension and type 2 diabetes mellitus, two dominant risk factors for CVD and mortality, and induces extensive arterial damage on coronary and peripheral limb arteries. Concurrently, the proven beneficial cardiovascular effects of red wine consumption, as an integrated part of the Mediterranean diet, have been substantially supported by the scientific literature. Given the conflicting literature results regarding the potential beneficial effects of low doses of alcohol, recommendations should be cautiously made in subjects at high-risk of CVD. The inconsistent evidence over the cardioprotective impact of alcohol drinking supports the minimization of consumption, and the possible advantages of wine intake should be unequivocally confined to a low to moderate intake.

## Figures and Tables

**Table 1:** The effects of high-dose ethanol versus low to moderate alcohol consumption on the above-considered diseases.

Cardiovascular Disease or Risk Factor	High Alcohol Consumption	Low to Moderate Doses of Ethanol
**Arrhythmias**	- Progressive atrial remodeling with a significant risk of developing atrial fibrillation if aged 30 years or above	- Consumption of only 12 g of alcohol per day enhances with 16% the risk of atrial fibrillation
	- Predispose to malignant ventricular arrhythmias and sudden cardiac death	
**Dilated Cardiomyopathy**	- A consumption of >80 g/day for more than 5 years favors the emergence of alcoholic cardiomyopathy	- No safe amounts of ethanol in alcoholic cardiomyopathy
**Hypertension**	- High alcohol consumption increases blood pressure at 13 h after consumption - High drinking frequency results in high prevalence of hypertension	- Emerging evidence indicates that small amounts of alcohol do not have any protective effect on blood pressure - Any drinking levels increases the risk of hypertension
**Atherosclerotic Vascular Disease**	- Heavy drinking increases the risk of mortality from coronary heart disease - Positively correlates with peripheral atherosclerotic plaque volume	- A dose–response relationship was documented between ethanol intake and diabetic lower extremity arterial disease
**Diabetes mellitus**	- Frequent alcohol drinking results in a higher prevalence of diabetes	- Consumption of more than 2 units of alcohol per day increases the risk while abstinence might help to prevent the development of diabetes

## Data Availability

Not applicable.
